# Chemokine receptor CCR2 is expressed by human multiple myeloma cells and mediates migration to bone marrow stromal cell-produced monocyte chemotactic proteins MCP-1, -2 and -3

**DOI:** 10.1038/sj.bjc.6600833

**Published:** 2003-03-18

**Authors:** I Vande Broek, K Asosingh, K Vanderkerken, N Straetmans, B Van Camp, I Van Riet

**Affiliations:** 1 Department of Hematology and Immunology, Vrije Universiteit Brussel (VUB), Laarbeeklaan 101, 1090 Brussels, Belgium; 2 Department of Hematology, Université Catholique de Louvain, Brussels, Belgium

**Keywords:** multiple myeloma, CCR2, chemokines, homing, bone marrow

## Abstract

The restricted bone marrow (BM) localisation of multiple myeloma (MM) cells most likely results from a specific homing influenced by chemotactic factors, combined with the proper signals for growth and survival provided by the BM microenvironment. In analogy to the migration and homing of normal lymphocytes, one can hypothesise that the BM homing of MM cells is mediated by a multistep process, involving the concerted action of adhesion molecules and chemokines. In this study, we report that primary MM cells and myeloma derived cell lines (Karpas, LP-1 and MM5.1) express the chemokine receptor CCR2. In addition, we found that the monocyte chemotactic proteins (MCPs) MCP-1, -2 and -3, three chemokines acting as prominent ligands for CCR2, are produced by stromal cells, cultured from normal and MM BM samples. Conditioned medium (CM) from BM stromal cells, as well as MCP-1, -2 and -3, act as chemoattractants for human MM cells. Moreover, a blocking antibody against CCR2, as well as a combination of neutralizing antibodies against MCP-1, -2 and -3, significantly reduced the migration of human MM cells to BM stromal cell CM. The results obtained in this study indicate the involvement of CCR2 and the MCPs in the BM homing of human MM cells.

Multiple myeloma (MM) represents a neoplastic B-cell disorder, characterised by a monoclonal and uncontrolled expansion of malignant plasma cells within the bone marrow (BM) compartment (Ludwig *et al*, 1999). Although MM cells primarily localise in the BM, they can also be found in the circulation, and the number of circulating cells increases at very advanced stages of disease. It can be assumed that these circulating MM cells are involved in disease spreading and must therefore have the potential to extravasate and home to the BM (Van Riet *et al*, 1998). In analogy to the migration and homing of normal lymphocytes, the BM homing of MM cells is likely to be mediated by specific mechanisms, involving the action of locally produced factors with chemotactic properties (Butcher and Picker, 1996). Chemokines are a family of small, structurally related cytokines with chemoattractant and activation properties involved in several types of inflammatory reactions (Oppenheim *et al*, 1991). They are characterised by the presence of a conserved cysteine motif near the N-terminal end. They can be classified into four distinct groups (C, CC, CXC and CX3C chemokines) based on the presence or absence of an amino-acid sequence separating the two first cysteines (Baggiolini *et al*, 1994; Premack and Shall, 1996). They are produced by a number of cell types, including leukocytes, endothelial cells, fibroblasts and stromal cells. Chemokines act on responsive cell types through G-protein coupled seven transmembrane receptors (Murphy *et al*, 1994). They have primarily been functionally related to leukocyte trafficking, but recent reports suggest their role in cancer development and progression as well (Strieter, 2001; Muller *et al*, 2001).

CCR2 is a chemokine receptor that is expressed on peripheral blood monocytes, as well as activated T cells, B cells and immature dendritic cells (Frade *et al*, 1997; Vecchi *et al*, 1999). Gene-targeted mice lacking CCR2 (*CCR2*−/− mice) exhibit defects in monocyte/macrophage trafficking to sites of inflammation (Kurihara *et al*, 1997; Boring *et al*, 1998; Peters *et al*, 2000). The known ligands for CCR2 include the monocyte chemotactic proteins (MCPs) MCP-1, -2 and -3 belonging to the family of CC chemokines (Mellado *et al*, 1998). They act as potent activators and chemoattractants for monocytes, basophils, eosinophils, T-lymphocyte subsets, dendritic cells and endothelial cells, but not neutrophils (Baggiolini *et al*, 1994; Salcedo *et al*, 2000). In addition, MCP-1 and -3 have shown antitumour activity by chemokine gene transfer in mouse models (Hoshino *et al*, 1995; Fioretti *et al*, 1998). MCP-1 has also been implicated in angiogenesis (Salcedo *et al*, 2000).

To date, very few data are available about the effects of chemokines on human MM cell migration (Woodliff and Epstein, 1999,^2000^). In this study, we analysed the functional expression of CCR2 on MM cell lines (HMCL) as well as primary MM cells from BM of MM patients. Our data demonstrate that HMCL, as well as primary MM cells, express CCR2. In addition, the MCPs, produced by BM stromal cells, felicitated migration responses, suggesting a potential contribution to the homing of MM cells to the BM microenvironment.

## MATERIALS AND METHODS

### Chemokines and antibodies

The human recombinant monocyte chemotactic proteins MCP-1, -2 and -3 were obtained from Biosource. Monoclonal antibodies (MoAb) against MCP-1, -2 and -3 were purchased from R&D. The CCR2 MoAb was a kind gift from Dr C Clement (Millennium Pharmaceuticals, Cambridge, MA, USA).

### Cell lines

Three well-characterised human MM cell lines (HMCL) (LP-1, Karpas and MM5.1) were selected for our experiments. They were kept in culture as described (Nilson, 1971; Okuno *et al*, 1991; Van Riet *et al*, 1997).

### Patient samples

BM samples from 28 MM patients (pts) (age 41–94 years, mean 66) were collected during standard diagnostic procedures. Each MM patient was diagnosed and staged according to the criteria of Durie and Salmon (1975). The study was approved by the local ethical committee. BM aspirates were obtained from the posterior iliac crest or sternum and collected in a heparinised syringe. Mononuclear cells (MNC) were separated by Ficoll density gradient centrifugation (Nycomed, Lucron, Gent, Belgium).

### MACS separation of primary MM cells

Primary MM cells were immunomagnetically separated using the magnetic cell sorting system (MACS) (Miltenyi Biotech Sanvertech, Bouchout, Belgium). MNC were incubated for 15 min at 4°C with MACS microbeads conjugated to a monoclonal mouse CD138 (syndecan-1) antibody (clone B-B4, isotype mouse IgG1). Cells were washed once in PBS supplemented with human albumin (4%), resuspended and separated on a column placed in the magnetic field of the MACS separator. CD138+ cells were retained and eluted as a positively selected cell fraction after removal of the column from the magnetic field. Cells were counted and viability was assessed with trypan blue. MACS purification produced a 98% pure primary MM cell population as determined by May – Grünwald – Giemsa-stained cytospin preparation.

### BM stromal cell culture and conditioned medium

MNC from BM samples obtained from MM patients and normal controls were cultured in 75 cm^2^ flasks (Nunc, VWR International, Leuven, Belgium) in RPMI supplemented with 12.5% foetal calf serum (FCS) and 12.5% horse serum at 37°C with 5% CO_2_. After 3–5 weeks of culture, a confluent layer was obtained and stromal cells were detached by trypsinisation. After one passage, established confluent cell layers were cultured for 5 days in serum-free medium (RPMI) and culture supernatant was harvested as conditioned medium (CM). CM was centrifuged to remove cell debris and frozen at −20°C until use. CM preparations from different cell cultures were prepared.

### RNA extraction, cDNA synthesis and reverse transcription polymerase chain reaction (RT–PCR)

Total RNA was extracted from cultured HMCL and cultured BM stromal cells from normal and MM BM samples using the RNeasy Mini Kit (Qiagen), according to the manufacturer's instructions. First-strand cDNA was generated from 5 *μ*g of total RNA with the SUPERSCRIPT™ Preamplification System (GIBCO BRL) according to the instructions made by the manufacturer. The amount of DNA corresponding to 1/10 of the cDNA obtained by reverse transcription was amplified with a PCR protocol in the presence of HotStarTaq Master Mix (Qiagen) and primer pairs (10 *μ*M) (GIBCO BRL) in a 25 *μ*l reaction mixture. The following primers were used for amplifying mRNA: CCR2 sense: 5′-TGG CTG TGT TTG CTT CTG TC-3′ and CCR2 antisense: 5′-TCT CAC TGC CCT ATG CCT CT-3′, (actin sense: 5′-TGC CTA TCC AGG CTG TGC TAT-3′ and actin antisense: 5′-GAT GGA GTT GAA GGT AGT TT-3′; MCP-1 sense: 5′-CTC AGC CAG ATG CAA TCA ATG C-3′ and MCP-1 antisense: 5′-CCT CAA GTC TTC GGA GTT TGG G-3′; MCP-2 sense: 5′-ATG CTG AAG CTC ACA CCC TTG CCC-3′ and MCP-2 antisense: 5′-CAG ATG CTT CAT GGA ATC CCT GAC C-3′; MCP-3 sense: 5′-CAG ATT TAT CAA TAA GAA AAT CCC-3′ and MCP-3 antisense: 5′-GTG CTT CAT AAA GTC CTG GAC CC-3′ (Dumoulin *et al*, 1999). The PCR profile consisted of an initial activation step of 15 min at 95°C, a 1 min initial denaturation at 94°C, followed by 30 cycles of 1 min denaturation at 94°C, 1 min annealing at 58, 55, 58, 68 and 54°C for CCR2, actin, MCP-1, -2 and -3, respectively, 2 min renaturation at 72°C and finally 10 min extension at 72°C. PCR products were analysed by electrophoresis in a 2% agarose gel, visualised by ethidium bromide and photographed (Kodak EDAS 290). The amplified mRNA was identified based upon the anticipated size by comparison with DNA ladder of known molecular sizes.

### Flow cytometry

Flow cytometry was used to assess CCR2 expression on the surface of HMCL and primary MM cells. For phenotyping the HMCL, cells were first incubated with mouse anti-human CCR2 MoAb (IgG_2a_) or control mouse IgG_2a_ (both at 10 *μ*g ml^−1^) for 30 min at 4°C. In the second step, cells were incubated with PE-conjugated goat anti-mouse IgG_2a_ antiserum (Southern Biotechnology InTec, Antwerpen, Belgium) for 30 min at 4°C. Cells were washed, resuspended in PBS and analysed on EPICS XL flow cytometer (Coulter Electronics Analis, Namur, Belgium). CCR2 expression on the surface of primary MM cells and normal plasma cells was evaluated by a double-staining procedure. MNC, isolated from BM samples of MM patients and normal controls by Ficoll gradient centrifugation, were incubated with CCR2 MoAb as described and a Cy-5-conjugated CD38 specific antibody (HIT2) (Becton Dickinson Erembodgem, Belgium). In the second step, cells were incubated with PE-conjugated goat anti-mouse IgG_2a_ antiserum (Southern Biotechnology). The fluorescence intensity was calculated from the fluorescence histogram and expressed as the fluorescence intensity ratio (FiR) (specific fluorescence/control fluorescence). A FiR value of more than 1.6 was considered positive.

### *In vitro* cell migration assay

Migration of HMCL and primary MM cells was assayed using Transwell™ cell culture inserts (Costar Corning Elscolab, Kruibekes, Belgium) as described previously (Vande Broek *et al*, 2001). CM from BM stromal cells or recombinant chemokines MCP-1, -2 and -3, diluted in 300 *μ*l RPMI 1640 medium in varying concentrations, were placed into 24-well culture plates (Costar Corning). Transwell™ inserts (6.5 mm diameter, 8 *μ*m pore size) were placed in each well and 1 × 10^5^ MM cells in 100 *μ*l RPMI were added to the upper chamber. Cells were allowed to migrate for 4 h at 37°C with 5% CO_2_, after which inserts were removed. The number of cells that transmigrated into lower wells was evaluated in two ways. For HMCL, a colorimetric assay with WST-8 was used (Cell Counting Kit-8 (Alexis)). WST-8 was added in each well and incubated for 4 h at 37°C. The absorbance of converted stain was then measured spectrophotometrically with a 96-well microplate reader (Ceres 900, Bio-Tek International Inc, Brussels, Belgium) at a wavelength of 450 nm with a reference wavelength of 620 nm. For primary MM cells, transmigrated cells were recovered from lower wells and counted with a FACSort flow cytometer (Becton Dickinson Erembodgem, Belgium). A known number of sphero blank calibration beads (Becton Dickinson Erembodgem, Belgium) were used as internal standard. Experiments were performed in triplicate and the mean with standard deviations (s.d.) was calculated. Migration responses were determined as the mean increase in cell migration as compared to control (spontaneous) migration. For migration-inhibition experiments, cells were preincubated for 30 min with a blocking CCR2 MoAb. In some experiments, cell migration was assayed in the presence of neutralising MoAbs against MCP-1, -2 and -3, which had been added to lower wells.

## RESULTS

### CCR2 is expressed by human MM cell lines (HMCL) and primary MM cells from patient BM samples

**Figure 1:**
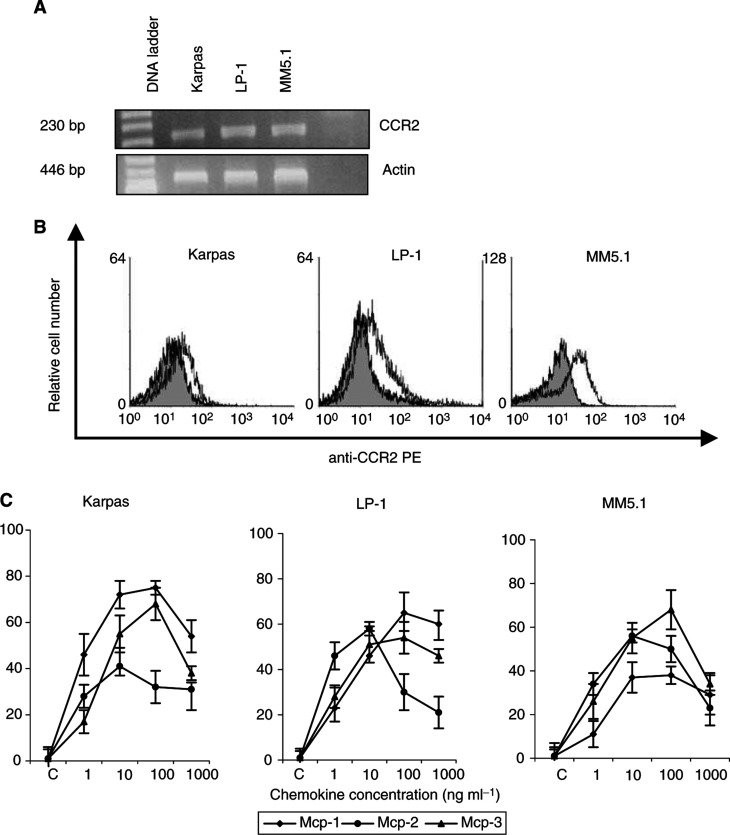
Expression of CCR2 chemokine receptor (**A** and **B**) and *in vitro* migration to MCP-1, -2 and -3 (C) by HMCL. CCR2 expression on HMCL was analysed by RT–PCR (**A**) and flow cytometry (**B**). RT–PCR analysis from total RNA extracted from HMCL with CCR2-specific primers showed a specific 230 bp PCR fragment in all three HMCL tested. Analysis of actin mRNA expression (446 bp PCR fragment) served as an internal control. Water was used as negative control. FACS analysis showed surface expression of CCR2 on HMCL. Results are shown as fluorescence histograms (open histogram: CCR2 expression; filled histogram: isotype-matched control antibody). Three HMCL were tested for their ability to migrate across 8 *μ*m pore-size polycarbonate filters in response to different MCP-chemokine concentrations as indicated (**C**). Values are expressed as the percentage increase of control migration to serum-free medium. Results shown are the mean and s.d. of three independent experiments.

**Figure 2:**
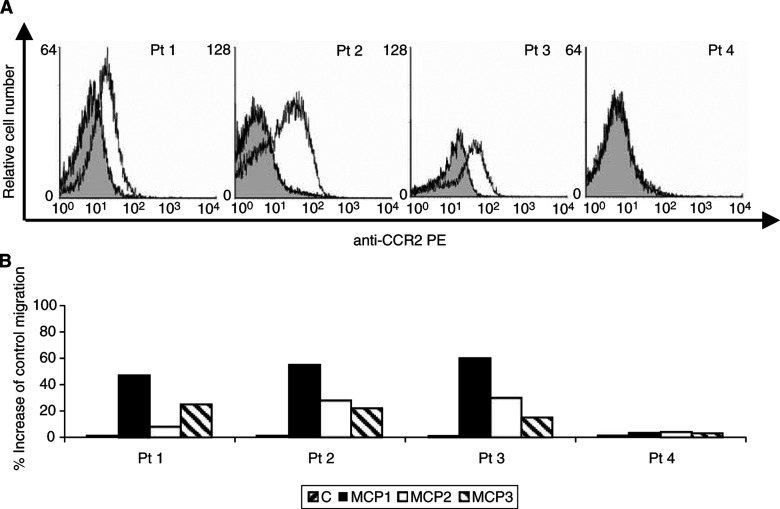
Expression and functionality of CCR2 on primary MM cells from BM samples: (**A**) FACS analysis of CCR2 on primary MM cells: Results are shown as fluorescence histograms. The open histogram shows CCR2 expression; the filled histogram represents the isotype-matched control antibody. Representative results of CCR2 expression from four different BM samples are shown. (**B**) *In vitro* cell migration to MCP-1, -2 and -3 was assayed using immunomagnetically isolated primary MM cells from four patient BM samples. Chemokines were used at 100 ng ml^−1^. The number of migrated cells was quantified by flow cytometry. Data represent the percentage increase of control migration. Representative values are shown for experiments with primary MM cells from four patient samples.

We first determined the expression of CCR2 by HMCL using RT – PCR and flow cytometry. RT – PCR of MM cell mRNA with specific primers for CCR2 showed the presence of CCR2 transcripts in all three HMCL tested (Figure 1A

). Flow cytometry with CCR2 MoAb demonstrated surface expression of CCR2 in all three HMCL (Figure 1B). Subsequently, we analysed surface expression of CCR2 on primary MM cells in BM samples from MM patients and normal controls using a two-colour staining method. FACS profiles revealed that CCR2 was expressed on primary MM cells in the majority (82%) of the patient samples tested (*n*=28). Results of CCR2 expression in primary MM cells from four BM samples are illustrated in Figure 2A

. Among the positive cases, the mean fluorescence intensity varied between 1.6 and 14.4. CCR2 was also found to be expressed on plasma cells from all normal BM samples tested (*n*=10) (data not shown).

### MCP-1, -2 and -3 act as chemoattractants for HMCL and primary MM cells

Since HMCL and primary MM cells express CCR2, the major chemokine receptor for the MCPs, it can be assumed that MCP-1, -2 and -3 function as chemoattractants for human MM cells. Therefore, we evaluated the migration of HMCL and primary MM cells in the presence of MCP-1, -2 and -3. Addition of MCP-1, -2 and -3 at concentrations from 1 to 1000 ng ml^−1^ to the lower compartments of the migration system resulted in a concentration-dependent stimulation of MM cell migration (Figure 1C). In the three HMCL, the maximal increase in cell migration to MCP-1 ranged between 38 and 75%, corresponding with 21–28% of the total number of cells in the upper compartment that actively migrated through the membrane into the lower compartment of the Transwell migration system. For MCP-2, the maximal increase in migration observed, varied between 41 and 56%, corresponding with 25–28% of the total number of input cells that migrated through the filter. In the presence of MCP-3, the maximal increase in migration observed, ranged between 54 and 68%, corresponding with 27–30% of the cells migrating to the lower compartment. Similar results were obtained using isolated primary MM cells from three MM patients (Pts 1 – 3), which were positive for CCR2. For all these patients, the most pronounced migration response was observed with MCP-1. In the presence of this chemokine, we observed an increase in cell migration between 48 and 60%, corresponding with 29–48% of cells migrating to the lower compartment. In one MM patient with CCR2-negative plasma cells (Pt 4), no significant migration response towards MCP-1, -2 or -3 could be observed (Figure 2).

### BM stromal cells express mRNA for MCP-1, -2 and -3

**Figure 3:**
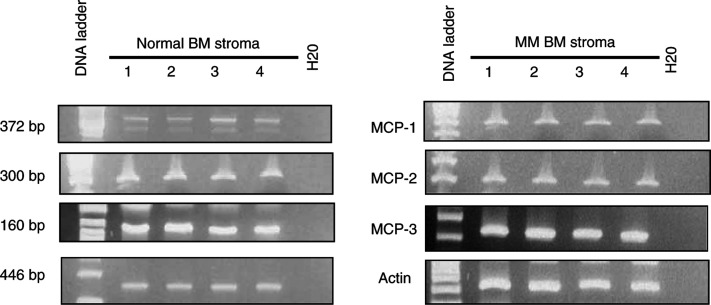
Expression of MCP-1, -2 and -3 in human BM stroma from MM patients and normal controls. Total RNA was isolated from cultured human BM stromal cells from MM patients and normal controls, and subjected to reverse transcription and PCR amplification for MCP-1, -2 and -3 using appropriate primers. The specific 372, 300 and 160 bp PCR fragments for MCP-1, -2 and -3, respectively, were detected in all normal and MM BM samples tested. Analysis of actin mRNA expression (446 bp PCR fragment) served as an internal control. Water was used as negative control.

Using specific primers for MCP-1, -2 and -3, we amplified PCR products of expected sizes (372, 300 and 160 bp, respectively) from cDNA of stromal cells, cultured from normal and MM BM samples (Figure 3

). Analysis of actin mRNA expression served as an internal control. These findings indicate that BM stromal cells from MM BM samples and normal controls produce the chemokines MCP-1, -2 and -3.

### Conditioned medium (CM) from BM stromal cells stimulates MM cell migration

**Figure 4:**
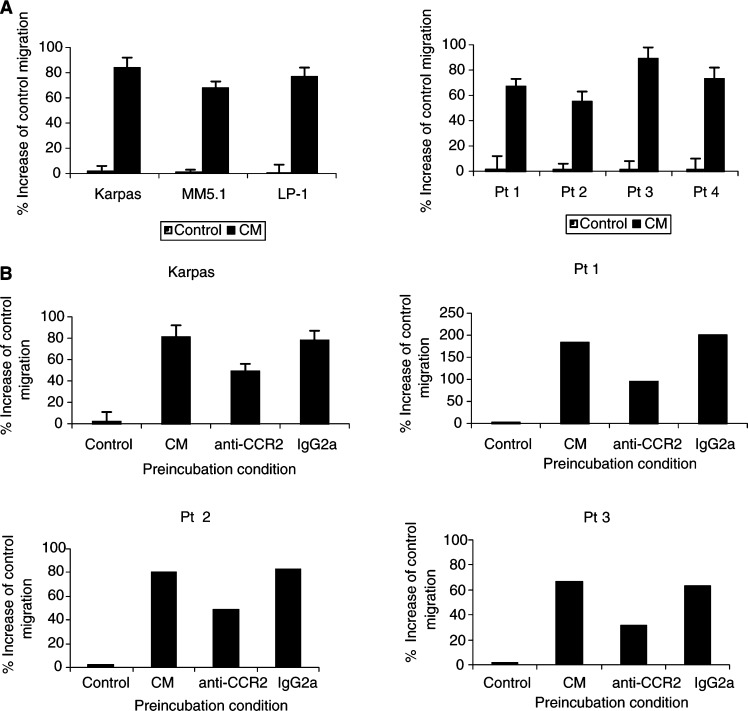
Effect of CM from BM stromal cells on human MM cell migration and inhibition by anti-CCR2 MoAb. *In vitro* cell migration was analysed by *in vitro* Transwell migration assay. BM CM was added to the lower compartment. Migration of three HMCL (Karpas, MM5.1 and LP-1) and primary MM cells from four MM patient samples was assayed. Results are expressed as the percentage increase of control migration to serum-free medium (**A**). For migration-inhibition experiments, MM cells were preincubated with a MoAb against the chemokine receptor CCR2 or a control antibody (IgG2a) prior to the migration assay. Results indicate the relative migration compared with control migration to CM and represent the mean value±s.d. of three experiments with Karpas cells. Representative values are also shown for experiments with primary MM cells from three MM patients (**B**).

We next determined whether supernatants from cultured BM stromal cells could stimulate MM cell migration. Migration of HMCL and primary MM cells was determined as described in Materials and Methods. CM was placed in the lower compartments of the migration system. As shown in Figure 4A

, the migration response of HMCL and primary MM cells was significantly enhanced (67 – 83 and 52 – 89% respectively, corresponding with 25 – 28 and 38 – 41% of migrating cells, respectively) when compared to control migration to serum-free RPMI medium. No differences were found in migration responses to CM from stromal cells, cultured from normal BM samples as compared to CM from MM BM samples (data not shown).

### CM-induced MM cell migration is inhibited by MoAbs against CCR2 and MCP-1, -2 and -3

**Figure 5:**
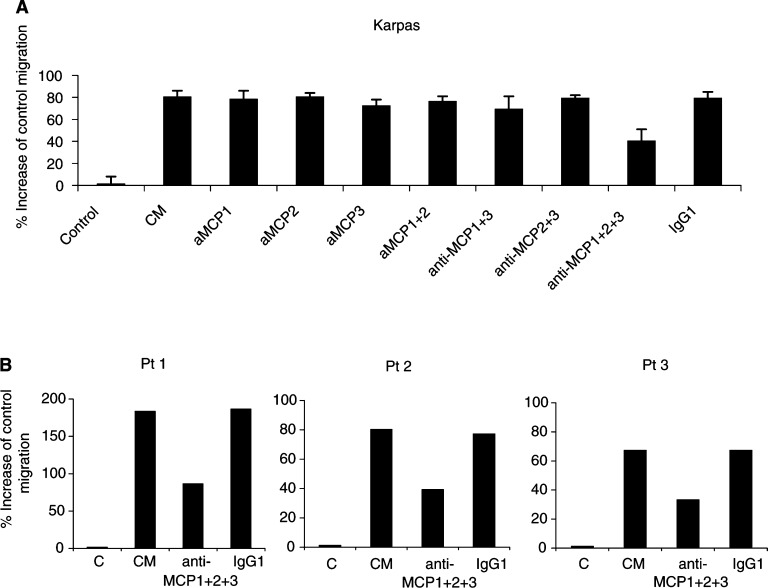
*In vitro* MM cell migration to CM is inhibited by anti-MCP MoAb. MoAb against MCP-1, -2 and -3 or control antibody (IgG1) was added together with CM to the lower compartment of the migration system. Results indicate the relative migration compared with control migration to CM and represent the mean value±s.d. of three experiments with Karpas cells (**A**). Representative values are shown for experiments with primary MM cells from three MM patients (**B**).

To determine whether the chemokines MCP-1, -2 and -3 produced by BM stromal cells are involved in the chemoattractive effect of BM CM, we performed migration experiments with MM cells towards BM CM in the presence of a blocking CCR2 MoAb or neutralising MCP MoAbs. MM cells were preincubated with CCR2 MoAb prior to the migration assay, and migration responses towards BM CM were analysed. In some experiments, MCP-1, -2 and -3 MoAbs were added to the lower compartment of the migration system together with CM. MM cell migration to BM was significantly inhibited by CCR2 MoAb up to 40% for Karpas cells and up to 52% for the patient samples (Figure 4B). The presence of one single MoAb against MCP-1, -2 or -3 did not significantly affect MM cell migration to BM CM, but in the presence of three MoAbs together against MCP-1, -2 and -3, MM cell migration was reduced up to 40% for Karpas cells and up to 50% for the isolated primary MM cells (Figure 5A,B

). These experiments clearly demonstrate that CM-induced MM cell migration involves MCP-1, -2 and -3 and that MM cell migration to MCPs occurs through CCR2.

### DISCUSSION

A striking feature of MM represents the selective localisation of malignant plasma cells in the BM. The mechanisms by which MM cells traffic to and accumulate in the BM are not fully understood. In analogy to the migration and homing of normal lymphocytes (Butcher and Picker, 1996), one can hypothesise that the BM homing of MM cells is mediated by a multistep process. First, circulating cells reversibly roll onto the endothelium, followed by a firm adhesion, transendothelial migration with passing through the basement membrane and finally migration to the extracellular matrix. Our group has demonstrated that murine 5T2 MM cells selectively migrate to the BM, the spleen and the liver, but only survive within the BM compartment and the spleen. So, the selective localisation of MM cells in the BM likely results from a combination of a selective homing and survival in the BM (Vanderkerken *et al*, 2000). In the same murine 5T model, we could also recently demonstrate that the BM homing of MM involves the adhesion receptor CD44v6 that mediates binding to BM endothelium (Asosingh *et al*, 2000). It is believed that homing not only requires specific cellular adhesion, but also depends on the interaction of locally produced chemoattractants with specific cell surface receptors (Butcher and Picker, 1996). Previously, we have demonstrated that laminin-1, a major component of the basement membrane, acts as a potent chemoattractant for human MM cells, indicating a key role for extravasation through the basement membrane (Vande Broek *et al*, 2001). In addition, MM cells were found to express the high-affinity laminin binding protein 67LR, which appeared to be upregulated by contact with the endothelium, indicating that during passage through the basement membrane, 67LR is contemporary upregulated allowing MM cells to be sensible to the chemoattractive properties of laminin-1, and so facilitating transendothelial migration. As a result of the broad expression of laminin-1 in basement membranes throughout the body, it is clear that this molecule on itself cannot be the only factor that determines the specificity of MM cell homing to the BM. We therefore focussed on other molecules with chemoattractive potential and started to analyse the particular role of chemokines in the BM homing process of MM cells. Chemokines represent a family of growing interest and recent findings indicate that these molecules are implicated in a complex network of signalisation between tumour cells and the microenvironment of the host (Amann *et al*, 1998; Ferrero *et al*, 1998).

We report here that HMCL and primary MM cells freshly isolated from the BM of MM patients express the chemokine receptor CCR2, as analysed by RT–PCR and/or flow cytometry. The chemokine receptor CCR2, together with CCR5, plays an important role in the recruitment of monocytes/macrophages and T cells in various inflammatory diseases, infection and arteriosclerosis (Kurihara *et al*, 1997; Boring *et al*, 1998). Previous reports have demonstrated that CCR2 is expressed on normal, mature B cells (Frade *et al*, 1997). Very recently, transcripts for this receptor were also identified in myeloma plasma cells, as demonstrated by cDNA arrays (De Vos *et al*, 2001). Our data show that CCR2 is also expressed in both, normal and malignant plasma cells, at the protein level. To our knowledge, no reports have been published yet describing the expression of the chemokine receptor CCR2 in other B-cell malignancies. Using Transwell™ migration assays, we could demonstrate that CCR2 expression on MM cells was functional since ligands of CCR2, that is MCP-1 as well as MCP-2 and -3, act as chemoattractants for human MM cells. Compared to control migration in the absence of chemoattractant, we noticed an increased cell migration response up to 80% for MCP-1, 60% for MCP-2 and 70% for MCP-3.

To investigate whether CCR2 and MCP-1, -2 and -3 are directly involved in the BM homing of MM cells, we first analysed the production of MCP-1, -2 and -3 by human stromal cells, cultured from normal and MM BM samples. A previous report described the production of MCP-1 by murine BM stromal cells (Xu *et al*, 1999). Moreover, MCP-2 mRNA has been identified in human BM stromal cells (Van Coillie *et al*, 1997). In this study, we demonstrated by RT–PCR, that BM stromal cells in MM samples express transcripts for MCP-1, -2 and -3. To determine the role of these chemokines in mediating MM cell migration to CM from cultured BM stromal cells, we performed *in vitro* migration assays in the presence of a blocking CCR2 MoAb. A reduction in cell migration up to 52% was observed. Additionally, *in vitro* migration assays were performed in the presence of neutralising MoAbs against MCP-1, -2 and -3. Interestingly, we observed no reduction of cell migration in the presence of one single MoAb or a combination of two MoAbs, but a clear reduction in MM cell migration was found when the three chemokines were neutralised simultaneously. It seems that preventing the binding of two MCP molecules to CCR2 still allows the third MCP to elucidate migration responses. Inhibition of the migration response to CM from BM stromal cells with a blocking CCR2 MoAb and neutralising MCP MoAbs indicates that MCP-1, -2 and -3 represent a major chemotactic activity for MM cells released by BM stroma. Moreover, the recent finding that IL-6, a major growth factor in MM, can upregulate the production of MCP-1 (Biswas *et al*, 1998) suggests that at least one of these chemokines is abundantly present in the BM microenvironment of MM patients. The fact that blocking of CCR2 binding to MCP-1, -2 and -3 does not induce complete inhibition of MM cell migration suggests that one or more additional chemoattractant(s) is (are) involved in the BM homing of MM cells. Recent data indicate that human MM cells express the chemokine receptor CXCR4 and that this receptor mediates *in vitro* migration of MM cells to SDF-1, a BM stromal-derived factor (Woodliff and Epstein, 1999). Moreover, it was found that CXCR4-positive, but not CXCR4-negative, MM cell lines home to the BM in SCID mice, suggesting the involvement of this particular receptor in the BM homing of MM cells as well (Woodliff *et al*, 2000). Another chemokine that might influence the migration behaviour of MM cells is MIP-1*α*. This chemokine was found to be secreted at elevated levels by BM cells of MM patients and has been related to osteoclast activation (Choi *et al*, 2000). Future experiments have to explore at which level SDF-1 and MIP-1*α* co-act with the MCPs to mediate the chemoattraction of MM cells to the BM.

The fact that CCR2 was also found to be expressed by normal plasma cells and its ligands were found to be secreted by normal stromal cells as well indicates that these receptor–ligand interactions might be involved in normal plasma cell homing as well. One study described that *CCR2*−/− mice do not show obvious haematological abnormalities, but the distribution and BM homing of normal plasma cells was not evaluated (Kurihara *et al*, 1997).

In conclusion, the results obtained in this study indicate that MM cells express CCR2 and that this chemokine receptor is functional. In addition, the monocyte chemotactic proteins MCP-1, -2 and -3, produced by BM stroma, act as chemoattractants for human MM cells and are involved in MM cell migration to BM stroma. These findings indicate a contribution of the chemokine receptor CCR2 to the homing of MM cells to the BM microenvironment.
